# Analysis of a multicenter registry on evaluation of transit-time flow in coronary artery disease surgery

**DOI:** 10.1016/j.xjon.2023.08.023

**Published:** 2023-09-21

**Authors:** Mojgan Laali, Olivier Bouchot, Olivier Fouquet, Pablo Maureira, Jean-Philippe Verhoye, Pierre Corbi, Charles-Henri David, Cosimo D'Alessandro, Pierre Demondion, Guillaume Lebreton, Pascal Leprince

**Affiliations:** a Thoracic and Cardiovascular Surgery Department, Sorbonne Université, APHP, Groupe hospitalier Pitié-Salpétrière, Institute of Cardiology, Paris, France; b Cardio-Thoracic and Vascular Surgery Unit, Hospital Center University, Dijon, France; c Cardiac Surgery, Angers University Hospital Center, Angers, France; d Cardiac Surgery Unit, Hospital Center, University de Nancy, Nancy, France; e Thoracic and Cardiovascular Surgery Department, Hospital Center, University Rennes, Rennes, France; f Cardio-Thoracic and Vascular Surgery Unit, Hospital Center, University Poitiers, Poitiers, France; g Cardio-Thoracic and Vascular Surgery Unit, CHU Nantes, Nantes, France

**Keywords:** multicenter registry, coronary artery surgery, transit-time flow measurement

## Abstract

**Objective:**

The Evaluation of Transit-Time Flow in Coronary Artery Disease Surgery (EFCAD) registry aims to assess the influence of transit-time flow measurement (TTFM) in daily practice.

**Methods:**

EFCAD is a prospective, multicenter study involving 9 centers performing TTFM during isolated coronary artery bypass grafting. Primary end point was occurrence and risk factors of major adverse cardiac events, including perioperative myocardial infarction, urgent postoperative coronary angiogram and/or revascularization, and hospital mortality. Secondary end points were rate of graft revision during surgery and factors affecting graft flow. We respected the limit values set by the experts: mean graft flow >15 mL/minute and pulsatility index ≤5.

**Results:**

Between May 2017 and March 2021, 1616 patients were registered in the EFCAD database. After review, 1414 were included for analyses. Of those, 1176 were eligible for primary end point analysis. Graft revision, mainly due to inadequate TTFM values, occurred in 2% (29 patients). The primary end point occurred in 46 (3.9%) patients, and it was related with left anterior descending artery graft flow ≤15 mL/minute (odds ratio, 3.64; *P* < .001). Graft flow was related with number of grafts (3 vs 1-2, β = −1.6; 4-6 vs 1-2, β = −4.1; *P* < .001; β > 0 indicates higher flow), and graft origin (aorta vs Y, β = 9.2; in situ left internal thoracic artery vs Y, β = 3.2; in situ right internal thoracic artery vs Y, β = 2.3; *P* < .001).

**Conclusions:**

Data from EFCAD study suggest that TTFM is reliable to evaluate graft flow, and acceptance of inadequate flow on left anterior descending artery anastomosis influence postoperative outcomes. In our opinion, TTFM assessment should be routinely used in coronary artery bypass procedures, even if interpretation depends on learning curves.

## Graphical Abstract

**Figure:**
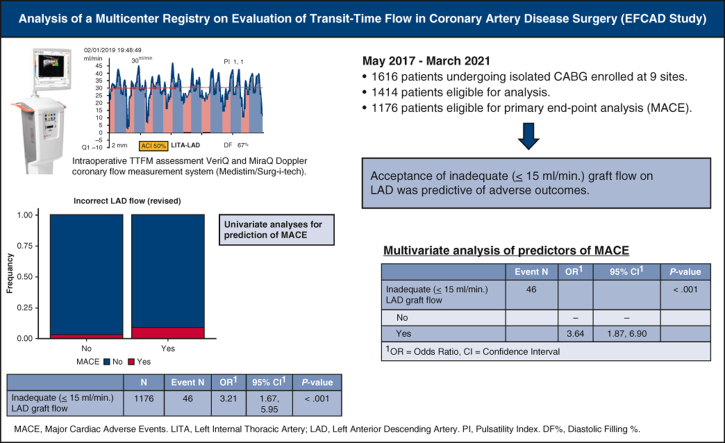

**Figure:**
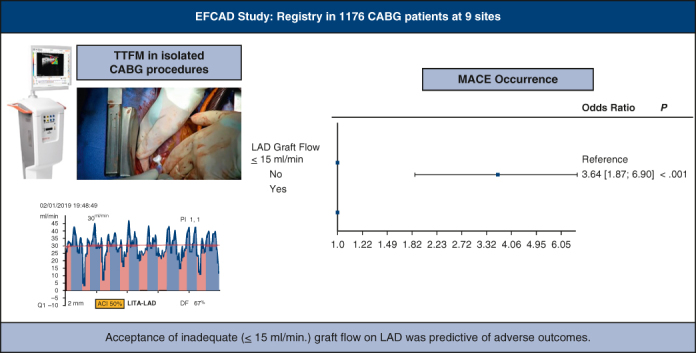
Inadequate (≤15 mL/minute) flow on LAD graft is a risk factor for the primary end point.

**Central Message**

Based a prospective multicenter registry focused on TTFM assessment in patients undergoing isolated CABG, acceptance of inadequate (≤15 mL/minute) graft flow on LAD was predictive of adverse outcomes.

**Perspective**

Data from EFCAD prospective multicenter registry suggest that TTFM is a reliable tool to evaluate graft flow and we found that postoperative adverse events are significantly higher in patients with inadequate (≤15 mL/minute) graft flow on LAD. Even if interpretation of TTFM assessment depends on learning curves and surgeon's commitment, it should be routinely adopted in CABG procedures.

In the context of coronary artery bypass graft (CABG) surgery, graft patency seems to play an important role in early and late postoperative outcomes.^1^^,^^2^ Technical errors could be among the factors, in addition to various factors influencing graft patency, and although rare, objective assessment of graft and anastomosis quality by intraoperative measurement of graft flow (GF) should be welcomed. Although several studies have led transit-time flow measurement (TTFM)^3^, ^4^, ^5^ to enter the guidelines,^6^ it has not yet routinely adopted by surgical community as a standard of care, with an estimated use rate of only 30% of procedures.^7^ Because it is time-consuming and the need for a learning curve to interpret the results have generally been put forward to explain the reluctance to use it, as well as the lack of results concerning its clinical influence, it remains controversial.^8^^,^^9^ This study aims to describe the influence of routine use of TTFM in our daily practice and to find any relationship between TTFM values and clinical outcomes.

## Patients and Methods

The Evaluation of Transit-Time Flow in Coronary Artery Disease Surgery (EFCAD) registry is a multi-institutional, prospective registry involving 9 French centers routinely performing CABG. The registry was originally designed to assess the association between postoperative outcomes and TTFM parameters measured with MiraQ device or VeriQ C devices (Medistim ASA). Preoperative patient data and outcomes were prospectively collected in an on-line database (EFCAD database), which received (French Commission of Information Technology and Freedom, Commission Nationale de l'Informatique et des Libertés) approval: 2060635 v 0 (May 3, 2017). An institutional review board grant was released by the ethical committee of the French Society of Thoracic and Cardiovascular Surgery (CERC-SFCTCV-2023-06-27_29236_Mojgan Laali), and the study received a research grant from Medistim ASA.

### Study Population

Between May 2017 and March 2021, 1616 patients undergoing primary isolated CABG were enrolled. Inclusion criteria were the need for isolated CABG and exclusion criteria were combined surgery, redo surgery, emergency surgery, and patients with very low ejection fraction (≤20%) for whom a temporary circulatory assist device was planned.

### Definitions and Outcomes

Primary end point of interest was the occurrence and the risk factors of major adverse cardiac events (MACE) at 30 postoperative days, including perioperative myocardial infarction, urgent postoperative coronary angiogram and/or revascularization, and hospital mortality. Secondary end points were rate of graft revision and factors influencing GF. Incomplete TTFM assessment, which means at least 1 graft not tested per patient, was also described. In case of revised graft, only TTFM values after revision were applied for analyses of GF and primary end point occurrence.

### TTFM Assessment

TTFM measurements were performed with the MiraQ or VeriQ C devices after crossclamp release, on partial cardiopulmonary bypass (CPB). The systolic blood pressure at the time of the measurements was at least 100 mm Hg. In case of off-pump procedures, assessment was carried out before protamine administration. The measurements were taken after all grafts were completed. The 2- or 3-mm probe was most commonly used. To obtain homogenous results, TTFM was assessed by respecting^7^ the following instructions: the acoustic coupling index must be >40% (displayed in green or yellow on the screen), indicating the accuracy of the ultrasonic conductivity; and the flow measurement was registered when mean flow, indicated by the red line, was constant and horizontal. The patency of the grafts was assessed using 3 variables: diastolic flow curve, mean flow, and pulsatility index (PI). Normally, the flow curve will show a small backflow during early systole and a predominantly forward flow during diastole.^7^ Cutoff values of TTFM assessment were mean GF > 15 mL/minute, PI ≤ 5, and diastolic flow ≥70% for left coronary bed and ≥50% for the right.^10^

### Statistical Analyses

Categorical variables were described as number (%) and continuous variables as median (interquartile range [IQR]). Risk factors of MACE and factors associated with an incomplete test were assessed using logistic regression model. Univariate analysis (*P* < .2) was first performed to select potential explanatory variables (patient's characteristics, surgical technique, and TTFM parameters) that were subsequently tested in multivariate model (backward variable selection based on *P* values) and presented as odds ratio (OR) with 95% CI. Factors associated with GF were assessed using linear mixed model with a random effect patient (several measurements for each patient). Univariate analysis (*P* < .2) was first performed to select potential explanatory variables (patient's characteristics, surgical technique, and other TTFM parameters) that were subsequently tested in multivariate model (backward variable selection based on *P* values) and presented as beta coefficients with 95% CI. Statistical analyses were performed using R Statistical Software version 4.1.0 (R Foundation for Statistical Computing).

## Results

### Patients

**Figure 1:**
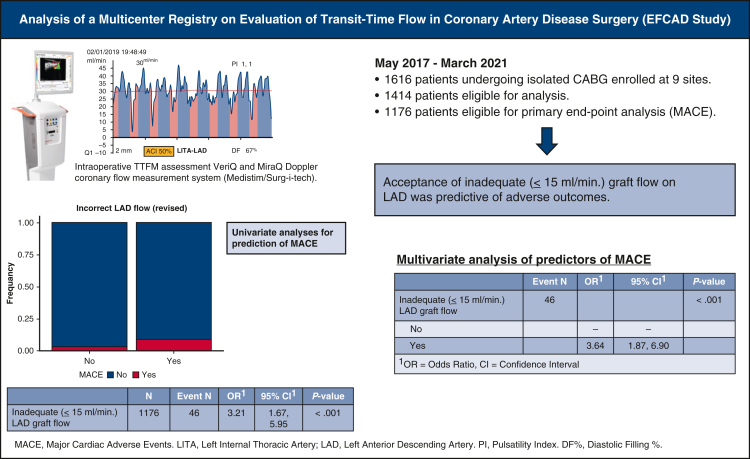
Graphical abstract. *LITA*, Left internal thoracic artery; *LAD*, left descending artery; *ACI*, acoustic coupling index; *PI*, pulsatility index; *DF*, diastollic filling; *TTFM*, transit-time flow measurement; *MACE*, major adverse cardiac event; *CABG*, coronary artery bypass grafting.

**Figure 2:**
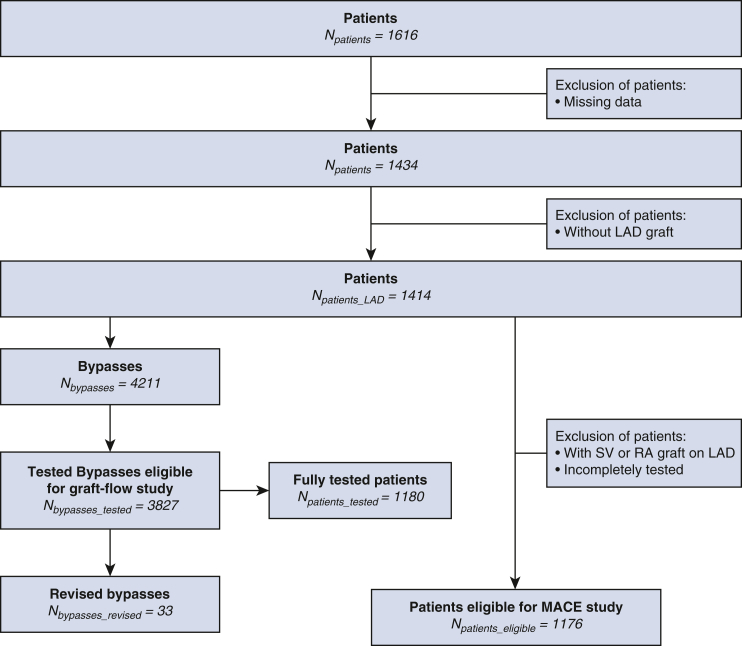
Flow chart with details of inclusions and eligibility of patients. *LAD*, Left descending artery; *SV*, saphenous vein; *RA*, radial artery.

One hundred eighty-two patients were excluded for missing data and 20 patients because they did not undergo left anterior descending artery (LAD) artery revascularization: after review, 1414 patients were eligible for analysis. Of those, 238 patients were excluded from primary end-point analysis because of incomplete TTFM assessment (n = 234 [17%]) or because they received a conduit other than an internal thoracic artery (ITA) on the LAD (n = 4 [0.3%]). Therefore, 1176 patients were eligible for primary end-point analysis. Figure 1 is the graphical abstract of the study. Figure 2 shows a flow chart with details of inclusions and eligibility of patients. Preoperative characteristics and operative data are summarized in Table 1.

**Table 1:** Preoperative characteristics, operative data, and postoperative outcomes (N = 1414)

Characteristic	Result
Preoperative characteristics	
Age (y)	67.82 (61.02-73.33)
Gender	
Female	205 (14.50)
Male	1209 (85.50)
Smoking, active or history of	596 (42.15)
History of smoking	421 (29.77)
Active smoking	175 (12.38)
Insulin-dependent diabetes	228 (16.12)
Hypertension	1007 (71.22)
Operative data	
Off-pump surgery	180 (12.73)
No. of distal anastomoses with data	
1	143 (10.11)
2	357 (25.25)
3	432 (30.55)
4	369 (26.10)
5	96 (6.79)
6	17 (1.20)
Total	4211 (100)
Graft tests	
Completely tested	1180 (83.45)
Incompletely tested	234 (16.55)
Total arterial revascularization	926 (65.49)
Total arterial revascularization with only ITAs	902 (63.79)
Grafts distribution	
No. of SV	
0	926 (65.49)
1	424 (29.99)
2	64 (4.53)
No. of RAs	
0	1387 (98.09)
1	24 (1.70)
2	3 (0.21)
No. of LITA	
0	12 (0.85)
1	889 (62.87)
2	487 (34.44)
3	26 (1.84)
No. of RITAs	
0	287 (20.30)
1	678 (47.95)
2	343 (24.26)
3	99 (7.00)
4	7 (0.50)
Graft BITA	1115 (78.85)
Graft origin distribution	
No. of in situ LITAs	
0	30 (2.12)
1	899 (63.58)
2	464 (32.81)
3	21 (1.49)
No. of Y-graft configurations	
0	607 (42.93)
1	390 (27.58)
2	315 (22.28)
3	95 (6.72)
4	7 (0.50)
No. of in situ RITAs	
0	1056 (74.68)
1	328 (23.20)
2	26 (1.84)
3	4 (0.28)
No. of AOs	
0	905 (64.0)
1	428 (30.27)
2	78 (5.52)
3	2 (0.14)
4	1 (0.07)
Intraoperative graft revision	29 (2.05)
No. of intraoperative graft revisions	
0	1385 (97.95)
1	25 (1.77)
2	4 (0.28)

Values are presented as median (interquartile range) or n (%). *ITAs*, Internal thoracic arteries; *SV*, saphenous vein; *RA*, radial artery; *LITA*, left internal thoracic artery; *RITA*, right internal thoracic artery; *BITA*, bilateral internal thoracic artery; *AO*, ascending aorta.

### TTFM Graft Assessment

TTFM was assessed for 3827 of 4211 (91%) grafts realized. Details of operative characteristics and TTFM parameters assessed by graft localization are resumed in Table E1. It is important to mention that because interpretation of the results in not easy, the surgeons were free to decide whether to revise the anastomosis or not based on the results of the measurements. There is no doubt that if any MACE occurred, it was then recorded in the database.

**Figure 3:**
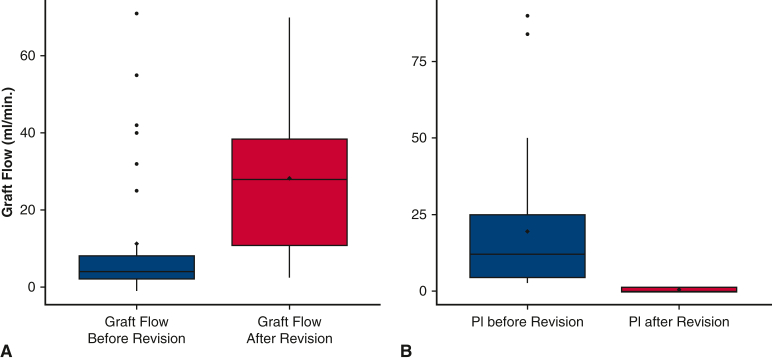
A, Graft flow before (median, 4.0 mL/minute; interquartile range [*IQR*], 2.0-8.0 mL/minute) and after revision (median, 28.0 mL/minute; IQR, 10.8-38.5) (n = 32; *P* .001). Data are presented as box and whisker dot plots with the upper and lower borders of the box representing the 25th and 75th percentile (upper and lower quartiles). The middle horizontal line represents the median and diamond dot the mean. The lower and upper whiskers represent the minimum and maximum values of nonoutliers. Extra dots represent outliers. B, Pulsatility index (*PI*) (median, 12.0; IQR, 4.5-25) before and after revision (median, 2.5; IQR, 2.05-4.23) (n = 33; *P* < .001). Data are presented as box and whisker dot plots with the upper and lower borders of the box representing the 25th and 75th percentile (upper and lower quartiles). The middle horizontal line represents the median and diamond dot the mean. The lower and upper whiskers represent the minimum and maximum values of nonoutliers. Extra dots represent outliers.

In our series, 33 grafts were intraoperatively revised in 29 patients (2%). Twenty-three grafts were revised for inadequate GF and PI values at the same time, 4 grafts for just inadequate GF, and 1 graft for inadequate PI; 5 grafts were revised despite correct values of both GF and PI. Eight hundred ninety-two grafts were not revised despite inadequate either GF and/or PI values. Revision was associated with significant GF improvement (median, 4.0; IQR, 2.0-8.0 vs median, 28.0; IQR, 10.8-38.5; *P* < .001) and PI reduction (median, 12.0; IQR, 4.5-25.0 vs median, 2.5; IQR, 2.05-4.23; *P* < .001) (Figure 3, *A* and *B*). Effectiveness of revised grafts according to inadequate flow and PI are presented in Tables E2 and E3.

### Primary End Point Outcome

Primary end point outcome occurred in 46 out of 1176 patients (OR, 3.9%; 95% CI, 2.9-5.2). Postoperative outcomes are detailed in Table 1. The primary end point occurred in 3 patients after revision:

- A left ITA (LITA) to LAD graft was revised because of low flow (8 mL/minute) and high PI (10). After revision, PI decreased to 2.9, but GF remained inadequate (2.4 mL/minute).
- A right RITA (RITA) to obtuse marginal graft was revised because of low flow (−1 mL/minute) and high PI (33). After revision, both GF and PI improved, but they remained inadequate (GF, 10 mL/minute, PI, 10).
- A LITA to obtuse marginal graft was revised because of low flow (3 mL/minute). After revision, GF improved to an adequate value of 48 mL/minute.

**Figure 4:**
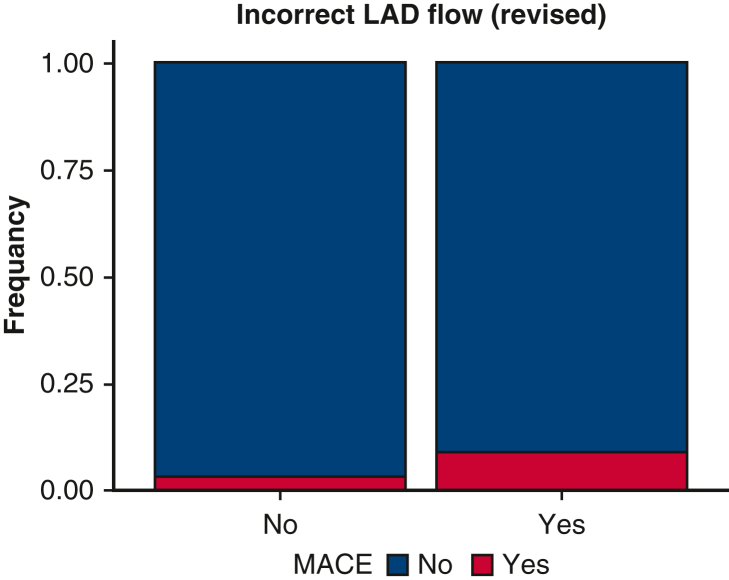
At univariate analysis, primary end point (major adverse cardiac event [*MACE*]) occurrence was higher in case of inadequate (≤15 mL/minute) flow on the left anterior descending artery (*LAD*) graft (odds ratio, 3.21; 95% CI, 1.67-5.95; *P* < .001).

**Figure 5:**
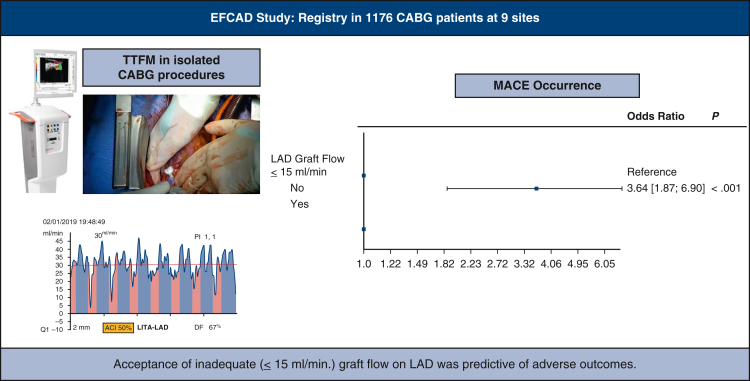
Inadequate (≤15 mL/minute) flow on left anterior descending artery graft is a risk factor for the primary end point. *EFCAD*, Evaluation of Transit-Time Flow in Coronary Artery Disease Surgery; *CABG*, coronary artery bypass grafting; *TTFM*, transit-time flow measurement; *PI*, pulsatility index; *LITA*, left internal thoracic artery; *LAD*, left descending artery; *DF*, diastollic filling.

We observed an increased occurrence of primary end point in patients needing graft revision, but this association did not reach a statistical significance (OR, 2.96; 95% CI, 0.69-8.84; *P* = .13). MACE occurrence was higher in case of inadequate (≤15 mL/minute) flow on the LAD graft (OR, 3.21; 95% CI, 1.67-5.95; *P* < .001) (Figure 4). In multivariate analysis, again acceptance of an inadequate flow (≤15 mL/minute) on the LAD graft was associated with adverse outcomes (OR, 3.64; 95% CI, 1.87-6.90; *P* < .001) (Figure 5). Other predictive factors of primary end point occurrence were off-pump surgery (OR, 3.13; 95% CI, 1.61-5.87; *P* = .001) and use of RITA as conduit for LAD graft (OR, 2.74; 95% CI, 1.38-5.22; *P* = .005). Analyses of risk factors for primary end point occurrence are detailed in Tables E4 and E5.

### GF

At multivariate analysis, GF was related to surgical technique, other TTFM parameters, and patient's profile. GF was significantly lower in case of revascularization with multiple sequential arterial grafts for each anastomosis, even if it remained in the normal range. A free-graft with a proximal anastomosis on the aorta showed the highest flow in comparison with a Y-configuration, followed by in situ ITA grafts: aorta versus Y, β = 9.2 (β > 0 = higher flow); in situ left ITA versus Y, β = 3.2; in situ RITA versus Y, β = 2.3; *P* < .001. Patient characteristics associated with high GF were male gender (β = 3.4; *P* = .009) and smoking status (β = 3.8; *P* < .001). The other TTFM parameters, such as PI (β = −0.62; *P* < .001) and diastolic filling % (β = 0.31; *P* < .001), showed a significant association with GF. Data concerning factors associated with GF are detailed in Tables E6 and E7.

### Incomplete TTFM Assessment

Complete assessment (all bypass tested vs at least 1 bypass not tested) of all grafts was achieved in 1180 (83%) patients. Exhaustiveness of Doppler measures was related, in multivariate analysis, to patient's characteristics and surgical technique. For example, revascularization with multiple sequential arterial grafts, especially in Y or T configuration, is more challenging because great care must be taken when handling the grafts to avoid inadvertent traction on the grafts and anastomotic tears, especially when doing measurements after crossclamp release, while the heartbeat is resuming.

An issue was also related to the design of the Doppler probe we used at the start of the study, which was solved with the handle-less model. Our study confirm that the number of distal anastomoses (NDA) (3 vs 1-2 = OR, 2.86; 4-6 vs 1-2 = OR, 3.03; *P* < .001) and NDA with RITA (2-4 vs 0-1 = OR, 1.87; *P* = .017) were associated with a higher probability incomplete assessment, whereas NDA with saphenous vein (1-2 vs 0 = OR, 0.25; *P* < .001), age (OR, 0.98; *P* = .007), and smoking status (OR, 0.43; *P* < .001) were associated with a lower probability of being not completely tested. The factors associated with incomplete testing are detailed in Tables E8 and E9.

## Discussion

The EFCAD study is a prospective, multicenter registry involving nine academic centers in France, with the aim of verifying the influence of the systematic use of TTFM in our daily practice to find any relationship between TTFM values and clinical outcomes.

### Clinical Influence of TTFM

European Society of Cardiology guidelines on myocardial revascularization highly recommend perioperative graft evaluation by Doppler control, which is also advocated by a recent expert consensus,^11^ TTFM has not yet routinely adopted by surgical community as a standard of care.^7^ Reluctance against routine TTFM use rely upon controversies on real need and clinical benefit of the technique. In comparative studies, Becit and colleagues^12^ and Bauer and colleagues^13^ reported significant encouraging results using TTFM; in the REgistry for QUaliity assESsment with ultrasound imaging and Transit-time flow measurement in cardiac bypass surgery (REQUEST) registry,^14^ 25% of patients required a change in surgical strategy guided by TTFM and ultrasonic imaging of the aorta, conduits, and grafts, resulting in reduction of in-hospital mortality and morbidity. However, the Graft Imaging to Improve Patency Randomized Controlled Trial (GRIIP RCT)^8^ and a subanalysis of Randomized On-Off Bypass (ROOBY) trial^9^ failed to demonstrate any influence of TTFM on 1- and 5-year clinical outcomes. These discrepancies concerning clinical evidence could be explained by several factors. First, occurrence of acute adverse events in contemporary coronary surgery is rare, ranging between 2% and 7% during the postoperative period^8^^,^^14^ and 12% at 5 years.^15^ In the EFCAD registry, the overall occurrence of MACE was 3.9%. A direct link between inadequate TTFM values and adverse postoperative outcome may be difficult to show because an impaired graft may have no immediate clinical influence, resulting in a silent postoperative course. As noted by Gaudino and colleagues,^1^ the relationship between graft patency and clinical outcomes is a complex process that could be influenced by competitive flow, persistent collateral flow, diabetes, quality of target vessels, and other factors. However, even if small series reported no clinical influence of graft occlusion, a large number of studies found a correlation between graft patency and patient outcomes.^1^ As we recently published,^16^ in a series with total arterial revascularization with ITAs, MACE occurrence was significantly reduced by half, from 6.9% to 3.3%, by adopting TTFM. Moreover, every adverse event was reduced, even without reaching statistical significance.

This positive effect could be explained by the fact that a technical problem concerning the conduit or graft anastomosis in a multiple sequential arterial technique can be more dramatic, given that blood perfusion of a large part of the myocardium often depends on the flow in a single conduit.

**Figure 6:**
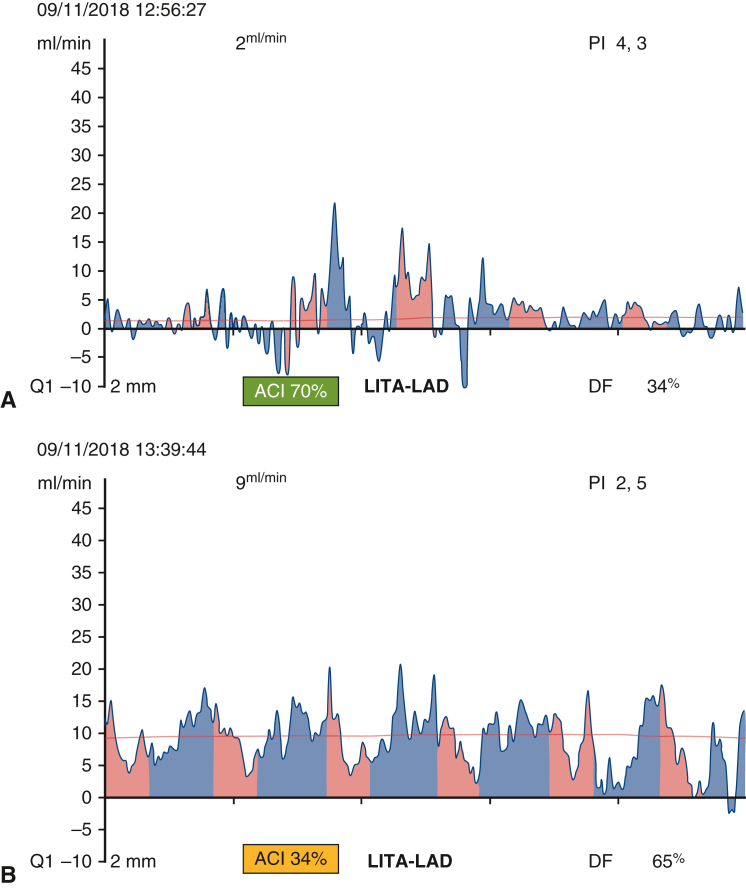
A, Transit-time flow measurement (*TTFM*) assessment of a malfunctioning left internal thoracic artery (*LITA*) to left anterior descending artery (*LAD*) graft before revision. B, TTFM assessment of a malfunctioning LITA to LAD graft after revision. *PI*, Pulsatility index; *DF*, diastollic filling; *ACI*, acoustic coupling index.

Studies have shown that, among TTFM parameters, PI was significantly associated with postoperative outcomes, either alone^17^ or in association with other clinical parameters.^3^ In the EFCAD registry, tolerance of an inadequate graft flow on LAD is strongly associated with adverse outcomes, probably because the aforementioned mechanisms are less likely to compensate an impaired LITA to LAD graft in presence of a severe proximal stenosis of the native coronary artery. Figure 6 shows TTFM recording before and after revision of a malfunctioning LITA to LAD graft. Even if PI is in a normal threshold, both GF value and waveform testify for an impaired graft (Figure 6, *A*). After revision, GF value increases and waveform recovers a normal shape (Figure 6, *B*).

### Adoption of TTFM

The surgeons who do not adopt TTFM assessment, believing on the 1 hand that the error rate is low, on the other hand, that doing the measurements is time-consuming and interpreting the results is not easy. It is right that TTFM assessment was associated with longer CPB times, but median extra time needed for measurement was only 3 minutes in our recently published study^16^ (median, 76.0 minutes; IQR, 62.0-91.2 minutes vs 79.0 minutes; IQR, 65.0-94.0 minutes; *P* = .042). Several studies confirm that graft revision is an infrequent event, reported to be undertaken in 3.3% to 5.7% of patients with abnormal TTFM values.^18^ In the REQUEST study,^14^ among 25% of patients requiring a change in surgical strategy guided by TTFM and epicardial ultrasonography, only 7.8% were solely related to the grafts. In the EFCAD registry, graft revision rate was even lower: 2% (per patient rate) with only 33 grafts revised out of a total of 3827 grafts tested, and 28 grafts revised out of 920 grafts with abnormal TTFM values. The EFCAD study showed that in 2 patients, after revision, improvement of TTFM values remained below adequate thresholds. Obviously, it is possible, and even probable, that anastomosis revision causes greater trauma to the anastomotic site and therefore higher risk of graft failure. Of course, we can understand that surgeons are reluctant to revise the grafts.

We confirm that interpretation of TTFM results is not easy and we know that, even if the threshold values and curves were defined for different types of grafts and revascularized vessels, standardization of TTFM findings is difficult because of large biologic variability among different patients, as well as within the same patient. The ability to correctly interpret TTFM findings develops with experience. On the other hand, we have to keep in mind that TTFM values are only useful and do not dictate the decision.

We emphasize that great care must be taken when handling the grafts to make the measurements to avoid inadvertent traction on the grafts and anastomotic tears, and this is another reason that restrains surgeons from using this technique. As underscored by Kieser and colleagues,^7^ assessment of a graft on the posterior or lateral wall could be not possible off-pump; in case of revascularization with multiple arterial sequential grafts, TTFM assessment could be difficult even while on partial CPB, which could also explain the 17% incomplete testing rate in EFCAD patients. Indeed, as reported in Table E1, whereas frequency of lateral or posterior grafts not tested ranged between 14% and 24%, only 4% of LAD grafts were not tested. We believe that, even with all these limitations, by gaining experience with this device, we can prevent a large number of unpleasant events. But surgeons who have not been exposed to TTFM technology cannot easily accord it the proper level of importance. Another consideration about the adoption of TTFM concerns training of residents in coronary surgery. A recent analysis^19^ showed that, by using TTFM with “appropriate supervision… residents can perform CABG with appropriate results, without compromising patient outcome.”

### GF

A recent meta-analysis confirmed that GF is lower in arterial than in venous grafts,^20^ which was also found in EFCAD univariate analysis (Table E6). Nevertheless, these results were irrespective of the graft origin, in situ or free-graft. Data from the EFCAD registry showed that highest flow is associated with free-grafts implanted on the aorta, followed by in situ ITA grafts and free-grafts implanted on a Y-configuration. These results are difficult to translate in a clinical setting: in the EFCAD registry, 79% of patients underwent a bilateral ITA revascularization and 64% received a total arterial revascularization with only ITAs (Table 1). Because graft flow was inversely related to the number of anastomoses, total arterial revascularization with multiple sequential grafts could be associated with lower flow per graft. So far, there is no evidence that anastomosis with more flow, when within the normal range, works better. By the way, TTFM is not here to verify the flow patterns, which depends on several factors, it is here to give us an objective assessment for the quality of grafts and anastomosis. As reported by Krasopoulos and colleagues,^21^ we also found a positive correlation between graft flow and male gender, probably because of larger diameter of coronary arteries and grafts; this may also explain the correlation between GF and smoking status, being more frequent in male patients.

### Limits

Due to its prospective design, the EFCAD study carries all the limits of a nonrandomized controlled trial, meaning lack of a control arm for comparison the results with or without Doppler control during CABG. Even if it has often been advocated, we believe that a randomized trial to check the effectiveness of Doppler graft control is not ethically possible and even desirable: An increasing amount of evidence is now available to confirm the association between TTFM values and graft patency, and more recently also between TTFM and clinical outcome.

## Conclusions

TTFM gives important and accurate intraoperative information about the status and patency of each individual graft. It enables technical problems such as kinked, twisted, or stenotic grafts to be diagnosed accurately, thereby allowing prompt revision of the constructed grafts before the patient leaves the operating room. Our data suggest that TTFM is a reliable intraoperative tool to evaluate graft flow and we found that postoperative adverse events are significantly higher in patients with an inadequate (≤15 mL/minute) flow on LAD graft (Figure 1). We have also noticed that this technology could be useful in university hospitals for residency training programs. Based on this study, we suggest that TTFM assessment should be routinely used in CABG procedures.

## Conflicts of Interest

Dr Laali has received speaker honoraria from Medistim ASA. All other authors reported no conflicts of interest.

The *Journal* policy requires editors and reviewers to disclose conflicts of interest and to decline handling manuscripts for which they may have a conflict of interest. The editors and reviewers of this article have no conflicts of interest.

## Figures and Tables

**Table E1:** Transit-time flow measurement (TTFM) graft assessment

Characteristic	LAD	LAD PROX	DIAG	DIAG2	OM1	OM2	OM3	PDA	RCA	PL	Total
	n = 1414	n = 17	n = 581	n = 58	n = 1065	n = 250	n = 15	n = 579	n = 89	n = 143	N = 4211
Operative characteristics by graft localization											
Not tested	58 (4.10)	4 (23.53)	49 (8.43)	10 (17.24)	85 (7.98)	58 (23.20)	3 (20.00)	83 (14.34)	4 (4.49)	27 (18.88)	381 (9.05)
No. of bypasses by center											
F06											
Tested	492 (37.79)	5 (29.41)	278 (47.85)	39 (67.24)	380 (35.68)	122 (48)	6 (40)	244 (42.14)	58 (65.17)	70 (48.95)	1694 (40.23)
Not tested	5 (0.35)	0 (0.00)	4 (0.69)	2 (3.45)	9 (0.84)	3 (1.20)	0 (0.00)	2 (0.33)	1 (1.12)	5 (3.50)	31 (0.74)
F11											
Tested	408 (28.85)	0 (0.00)	25 (4.30)	1 (1.72)	236 (22.16)	5 (2.00)	0 (0.00)	85 (14.68)	12 (13.48)	20 (13.99)	792 (18.81)
Not tested	24 (1.70)	0 (0.00)	9 (1.55)	0 (0.00)	28 (2.63)	1 (0.4)	0 (0.00)	3 (0.52)	1 (1.12)	0 (0.00)	66 (1.57)
F01											
Tested	344 (24.33)	8 (47.01)	194 (33.82)	5 (8.62)	287 (26.94)	64 (25.60)	6 (40.00)	143 (24.70)	5 (5.62)	20 (13.99)	1076 (25.55)
Not tested	29 (2.00)	4 (23.53)	36 (6.20)	8 (13.79)	47 (4.41)	54 (21.60)	3 (20.00)	78 (13.47)	2 (2.24)	22 (15.38)	283 (6.72)
F08											
Tested	73 (5.16)	0 (0.00)	24 (4.13)	2 (3.45)	53 (4.98)	1 (0.40)	0 (0.00)	15 (2.59)	7 (7.89)	1 (0.70)	176 (4.18)
Not tested	0 (0.00)	0 (0.00)	0 (0.00)	0 (0.00)	0 (0.00)	0 (0.00)	0 (0.00)	0 (0.00)	0 (0.00)	0 (0.00)	0 (0.00)
F18											
Tested	28 (1.98)	0 (0.00)	6 (1.03)	1 (1.72)	18 (1.69)	1 (0.40)	0 (0.00)	3 (0.52)	3 (3.37)	0 (0.00)	60 (1.44)
Not tested	0 (0.00)	0 (0.00)	0 (0.00)	0 (0.00)	0 (0.00)	0 (0.00)	0 (0.00)	0 (0.00)	0 (0.00)	0 (0.00)	0 (0.00)
F02											
Tested	5 (0.35)	0 (0.00)	1 (0.17)	0 (0.00)	4 (0.37)	0 (0.00)	0 (0.00)	0 (0.00)	0 (0.00)	1 (0.70)	11 (0.26)
Not tested	0 (0.00)	0 (0.00)	0 (0.00)	0 (0.00)	0 (0.00)	0 (0.00)	0 (0.00)	0 (0.00)	0 (0.00)	0 (0.00)	0 (0.00)
F03											
Tested	4 (0.28)	0 (0.00)	3 (0.52)	0 (0.00)	2 (0.19)	0 (0.00)	0 (0.00)	3 (0.52)	0 (0.00)	0 (0.00)	12 (0.28)
Not tested	0 (0.00)	0 (0.00)	0 (0.00)	0 (0.00)	1 (0.09)	0 (0.00)	0 (0.00)	0 (0.00)	0 (0.00)	0 (0.00)	1 (0.02)
F10											
Tested	2 (0.14)	0 (0.00)	1 (0.17)	0 (0.00)	1 (0.09)	0 (0.00)	0 (0.00)	2 (0.34)	0 (0.00)	0 (0.00)	6 (0.14)
Not tested	0 (0.00)	0 (0.00)	0 (0.00)	0 (0.00)	0 (0.00)	0 (0.00)	0 (0.00)	0 (0.00)	0 (0.00)	0 (0.00)	0 (0.00)
F17											
Tested	1 (0.07)	0 (0.00)	1 (0.17)	0 (0.00)	0 (0.00)	0 (0.00)	0 (0.00)	1 (0.17)	0 (0.00)	0 (0.00)	3 (0.07)
Not tested	0 (0.00)	0 (0.00)	0 (0.00)	0 (0.00)	0 (0.00)	0 (0.00)	0 (0.00)	0 (0.00)	0 (0.00)	0 (0.00)	0 (0.00)
Graft											
LITA	1183 (83.66)	15 (88.24)	439 (75.56)	20 (34.48)	208 (19.53)	67 (26.80)	2 (13.33)	5 (0.86)		2 (1.40)	1941 (46.09)
RA	1 (0.07)		3 (0.52)		8 (0.75)	1 (0.40)		11 (1.90)	5 (5.62)	1 (0.70)	30 (0.71)
RITA	227 (16.05)	2 (11.76)	90 (15.49)	31 (53.45)	795 (74.65)	168 (67.20)	11 (73.33)	274 (47.32)	22 (24.72)	69 (48.25)	1689 (40.11)
SV	3 (0.21)		49 (8.43)	7 (12.07)	54 (5.07)	14 (5.60)	2 (13.33)	289 (49.91)	62 (69.66)	71 (49.65)	551 (13.08)
Graft origin											
AO	14 (0.99)		54 (9.29)	8 (13.79)	70 (6.57)	16 (6.40)	2 (13.33)	294 (50.78)	63 (70.79)	72 (50.35)	593 (14.08)
In situ LITA	1171 (82.81)	15 (88.24)	426 (73.32)	18 (31.03)	189 (17.75)	64 (25.60)	2 (13.33)	3 (0.52)		2 (1.40)	1890 (44.88)
In situ RITA	127 (8.98)		20 (3.44)	7 (12.07)	186 (17.46)	22 (8.80)		13 (2.25)	14 (15.73)	3 (2.10)	392 (9.31)
Y-graft	102 (7.21)	2 (11.76)	81 (13.94)	25 (43.10)	620 (58.22)	148 (59.20)	11 (73.33)	268 (46.29)	10 (11.24)	66 (46.15)	1333 (31.66)
In situ ITA								1 (0.17)	2 (2.25)		3 (0.07)
Revised graft	15 (1.07)	0 (0.00)	4 (0.70)	0 (0.00)	6 (0.57)	1 (0.40)	0 (0.00)	7 (1.21)	0 (0.00)	0 (0.00)	33 (0.79)

	LAD	LAD PROX	DIAG	DIAG2	OM1	OM2	OM3	PDA	RCA	PL	Total
	n = 1356	n = 13	n = 532	n = 48	n = 980	n = 192	n = 12	n = 495	n = 83	n = 116	N = 3827
TTFM parameters by graft localization											
Graft flow	28 (19-43)	24 (20-42)	26 (17-40)	24 (16-35)	25 (16-37)	24 (15-36)	32 (14-34)	26 (16-40)	28 (16-44)	23 (16-37)	26 (17-40)
Missing			1								1
Pulsatility index	2.40 (1.80-3.00)	2.00 (1.50-2.60)	2.30 (1.70-3.00)	2.60 (1.85-3.40)	2.30 (1.70-3.10)	2.20 (1.60-3.20)	2.75 (1.53-3.85)	2.40 (1.60-3.55)	2.30 (1.60-3.10)	2.30 (1.70-3.73)	2.30 (1.70-3.10)
Missing	1		2	1							4
Diastolic filling (%)	72 (66-76)	72 (66-79)	70 (64-76)	65 (56-73)	67 (61-72)	65 (59-72)	66 (60-70)	62 (55-67)	56 (48-64)	64 (57-70)	68 (62-74)
Missing	48		31	3	40	11		23	8	3	167
Revised graft		0 (NA)		0 (NA)			0 (NA)		0 (NA)	0 (NA)	
LITA	11 (73.33)		3 (75.00)		2 (33.33)						16 (48.48)
RITA	4 (26.67)		1 (25.00)		4 (66.67)	1 (100.00)		4 (57.14)			14 (42.42)
SV								3 (42.86)			3 (9.09)
Revised graft by Center											
F06	12 (80.00)		4 (100)		5 (83.33)	1 (100)		5 (71.43)			27 (81.82)
F11	1 (6.67)										1 (3.03)
F01	1 (6.67)							2 (28.57)			3 (9.09)
F08	1 (6.67)				1 (16.67)						2 (6.06)
F018											
F02											
F03											
F10											

Values are presented as median (interquartile range) or n (%). *LAD*, Left Descending Artery; *LAD PROX*, proximal sequential graft on LAD; *DIAG*, diagonal artery; *DIAG2*, second sequential graft on diagonal artery; *OM1*, first sequential or terminal graft on obtuse marginal artery; *OM2*, second sequential graft on obtuse marginal artery; *OM3*, third sequential graft on obtuse marginal artery; *PDA*, posterior descending artery; *RCA*, right coronary artery; *PL*, postero-lateral artery; *LITA*, left internal thoracic artery; *RITA*, right internal thoracic artery; *SV*, saphenous vein.

**Table E2:** Characteristics of grafts with inadequate measurements and without revision

Flow	Pulsatility index	Unrevised	Revised
Adequate	Adequate	2895	5
Adequate	Inadequate	90	1
Adequate	Missing	1	0
Inadequate	Adequate	640	4
Inadequate	Inadequate	161	23
Missing	Missing	354	0
Inadequate	Missing	1	0
Missing	Adequate	3	0

**Table E3:** Graft characteristics by type of inadequate measure

Characteristic	By type of inadequate measure				Total	
	Flow: adequate and PI: inadequate (n = 90)	Flow: inadequate and PI: adequate (n = 640)	Flow: inadequate and PI: inadequate (n = 161)	Flow: inadequate and PI: NA (n = 1)	Non-missing; missing	N = 892
Localization					892; 0	
DIAG	4 (4.44)	93 (14.53)	18 (11.18)	0 (0.00)		115 (12.89)
DIAG2	1 (1.11)	9 (1.41)	2 (1.24)	1 (100.00)		13 (1.46)
L1	17 (18.89)	171 (26.72)	48 (29.81)	0 (0.00)		236 (26.46)
L2	3 (3.33)	38 (5.94)	13 (8.07)	0 (0.00)		54 (6.05)
L3	0 (0.00)	3 (0.47)	1 (0.62)	0 (0.00)		4 (0.45)
LAD	34 (37.78)	200 (31.25)	34 (21.12)	0 (0.00)		268 (30.04)
LADPROX	0 (0.00)	2 (0.31)	0 (0.00)	0 (0.00)		2 (0.22)
PDA	22 (24.44)	89 (13.91)	31 (19.25)	0 (0.00)		142 (15.92)
PL	5 (5.56)	21 (3.28)	8 (4.97)	0 (0.00)		34 (3.81)
RCA	4 (4.44)	14 (2.19)	6 (3.73)	0 (0.00)		24 (2.69)
Graft					892; 0	
LITA	34 (37.78)	291 (45.47)	57 (35.40)	1 (100.00)		383 (42.94)
RA	0 (0.00)	2 (0.31%)	0 (0.00%)	0 (0.00)		2 (0.22)
RITA	20 (22.22)	299 (46.72)	72 (44.72)	0 (0.00)		391 (43.83)
SV	36 (40.00)	48 (7.50)	32 (19.88)	0 (0.00)		116 (13.00)
Graft origin					892; 0	
Y	18 (20.00)	257 (40.16)	60 (37.27)	0 (0.00)		335 (37.56)
AO	36 (40.00)	48 (7.50)	29 (18.01)	0 (0.00)		113 (12.67)
In situ ITA	0 (0.00)	0 (0.00)	1 (0.62)	0 (0.00)		1 (0.11)
In situ LITA	34 (37.78)	280 (43.75)	51 (31.68)	1 (100.00)		366 (41.03)
in situ RITA	2 (2.22)	55 (8.59)	20 (12.42)	0 (0.00)		77 (8.63)
Graft flow	26 (21-33)	12 (9-14)	8 (4-12)	8 (8-8)	892; 0	12 (9-14)
PI	7.0 (5.4-11.0)	2.9 (2.1-3.8)	7.8 (6.2-13.3)	NA (NA-NA)	891; 1	3.6 (2.5-5.3)
Unknown	0	0	0	1		1
Diastolic filling (%)	58 (48-67)	66 (59-73)	49 (28-62)	NA (NA-NA)	854; 38	63 (54-71)
Unknown	1	32	4	1		38

Values are presented as n (%) or median (interquartile range). *PI*, Pulsatility index; *NA*, not available; *DIAG*, diagonal artery; *DIAG2*, second sequential graft on diagonal artery; *LAD*, left descending artery; *LAD PROX*, proximal sequential graft on LAD; *PDA*, posterior descending artery; *PL*, postero-lateral artery; *RCA*, right coronary artery; *LITA*, left internal thoracic artery; *RA*, radial artery; *RITA*, right internal thoracic artery; *SV*, saphenous vein; *AO*, ascending aorta; *PI*, pulsatility index; *ITA*, internal thoracic artery; *OM1*, first sequential or terminal graft on obtuse marginal artery; *OM2*, second sequential graft on obtuse marginal artery; *OM3*, third sequential graft on obtuse marginal artery.

**Table E4:** Predictive factors of primary end point occurrence (ie, major adverse cardiac event [MACE])

Characteristic	N	Event n	Odds ratio	95% CI	*P* value
Univariate analyses for prediction of MACE					
Age	1176	46	1.01	0.98-1.05	.38
Gender	1176	46			.28
Female			–	–	
Male			0.65	0.32-1.47	
Smoking status, active or history of	1176	46	1.03	0.57-1.86	.92
History of smoking	1176	46	0.69	0.34-1.32	.27
Active smoking	1176	46	1.80	0.83-3.57	.13
Insulin-dependent diabetes	1176	46	1.44	0.67-2.84	.34
Hypertension	1176	46	1.04	0.55-2.07	.91
Off-pump surgery	1176	46	3.23	1.69-5.99	<**.001**
No. of distal anastomoses, total	1176	46			.27
1-2			–	–	
3			1.68	0.84-3.41	
4-6			1.03	0.47-2.23	
No. of SV	1176	46			.54
0			–	–	
1-2			0.82	0.43-1.51	
No. of LITA	1176	46			>.99
0-1			–	–	
2-3			1.00	0.52-1.85	
No. of RITA	1176	46			.90
0-1			–	–	
2-4			0.96	0.46-1.85	
LAD graft	1176	46			**.007**
LITA			–	–	
RITA			2.52	1.30-4.69	
Last LAD flow	1176	46	0.99	0.98-1.01	.35
Inadequate Last LAD flow	1176	46	3.21	1.67-5.95	**<.001**
Revision	1176	46	2.96	0.69-8.84	.13
Any Inadequate flow	1176	46	1.12	0.60-2.02	.72
Any Inadequate PI	1176	46	1.22	0.54-2.46	.61
Any Inadequate diastolic filling (%)	1176	46	1.12	0.60-2.23	.73
At least 1 Inadequate measure	1176	46	1.37	0.66-3.20	.41

Figures in boldface are statistically significant. *SV*, Saphenous vein; *LITA*, left internal thoracic artery; *RITA*, right internal thoracic artery; *LAD*, left descending artery; *PI*, pulsatility index.

**Table E5:** Multivariate analysis of predictors of major adverse cardiac events

Characteristic	Event n	Odds ratio	95% CI	*P* value
Off-pump surgery	46			**.001**
No		–	–	
Yes		3.13	1.61-5.87	
LAD graft	46			**.005**
LITA		–	–	
RITA		2.74	1.38-5.22	
Inadequate last LAD flow	46			**<.001**
No		–	–	
Yes		3.64	1.87-6.90	

Figures in boldface are statistically significant. *LAD*, Left descending artery; *LITA*, left internal thoracic artery; *RITA*, right internal thoracic artery.

**Table E6:** Univariate analyses for graft flow∗

Characteristic	N	Beta	95% CI	*P* value
Age	3827	−0.03	−0.13 to 0.06	.52
Gender	3827			**.006**
Female		–	–	
Male		3.7	1.1-6.3	
Smoking status, active or history of	3827	4.9	3.1-6.7	**<.001**
History of smoking	3827	4.5	2.5-6.5	**<.001**
Active smoking	3827	2.3	−0.43 to 5.0	.10
Insulin-dependent diabetes	3827	0.19	−2.2 to 2.6	.88
Hypertension	3827	0.49	−1.5 to 2.5	.63
Off-pump surgery	3827	−0.43	−3.1 to 2.2	.75
No. of distal anastomoses, total	3827			**<.001**
1-2		—	—	
3		−1.8	−4.1 to 0.52	
4-6		−5.1	−7.3 to −2.9	
Graft revision	3818	−2.3	−9.4 to 4.8	.53
Graft	3827			**<.001**
LITA		—	—	
RA		1.8	−5.6 to 9.2	
RITA		−4.0	−5.3 to −2.8	
SV		1.9	0.09-3.8	
Graft origin	3827			**<.001**
Y		–	–	
AO		8.0	6.0-10	
In situ LITA		5.5	4.1-6.9	
In situ RITA		4.0	1.6-6.4	
PI, revised	3824	−0.89	−1.0 to −0.74	**<.001**
Diastolic filling %	3659	0.35	0.30-0.40	**<.001**
Localization	3827			**<.001**
Not LAD		–	–	
LAD		2.7	1.5-3.9	
Localization	3827			.17
Not LADPROX		–	–	
LADPROX		7.6	−3.1 to 18	
Localization	3827			.20
Not DIAG		–	–	
DIAG		1.1	−0.60 to 2.9	
Localization	3827			.28
Not DIAG 2		–	–	
DIAG 2		−3.1	−8.7 to 2.5	
Localization	3827			**<.001**
Not OM1		–	–	
OM1		−2.5	−3.8 to −1.1	
Localization	3827			**.047**
Not OM2		–	–	
OM2		−2.9	−5.7 to −0.04	
Localization	3827			.95
Not OM3		–	–	
OM3		0.35	−11 to 11	
Localization	3827			.49
Not PDA		–	–	
PDA		−0.63	−2.4 to 1.2	
Localization	3827			.86
Not RCA		–	–	
RCA		0.39	−3.9 to 4.7	
Localization	3827			.10
Not PL		–	–	
PL		−3.0	−6.6 to 0.61	

Figures in boldface are statistically significant. *LITA*, Left internal thoracic artery; *RA*, radial artery; *RITA*, right internal thoracic artery; *SV*, saphenous vein; *AO*, ascending aorta; *PI*, pulsatility index; *LAD*, left descending artery; *LAD PROX*, proximal sequential graft on LAD; *DIAG*, diagonal artery; *DIAG 2*, second sequential graft on diagonal artery; *OM1*, first sequential or terminal graft on obtuse marginal artery; *OM2*, second sequential graft on obtuse marginal artery; *OM3*, third sequential graft on obtuse marginal artery; *PDA*, posterior descending artery; *RCA*, right coronary artery; *PL*, postero-lateral artery.

∗ Because several measurements are given for each patients, a mixed-model was used, with a random effect according to the patient. This allows to take into account that some patient-based dependence (for 2 grafts from the same patient, some of the variability can be explained by the fact that both are from the same patient).

**Table E7:** Multivariate analysis of graft flow∗

Characteristic	Beta	95% CI	*P* value
Gender			**.009**
Female	–	–	
Male	3.4	0.85-6.0	
Smoking status, active or history of			**<.001**
No	–	–	
Yes	3.8	2.0-5.7	
No. of distal anastomoses, total			**<.001**
1-2	–	–	
3	−1.6	−3.9 to 0.66	
4-6	−4.1	−6.3 to −1.9	
Graft origin			**<.001**
Y	–	–	
AO	9.2	7.1-11	
In situ LITA	3.2	1.8-4.7	
In situ RITA	2.3	−0.10 to 4.7	
PI, revised	−0.62	−0.77 to −0.46	**<.001**
Diastolic filling (%)	0.31	0.25-0.36	**<.001**

Figures in boldface are statistically significant. *AO*, Ascending aorta; *LITA*, left internal thoracic artery; *RITA*, right internal thoracic artery; *PI*, pulsatility index.

∗ Variables associated to the outcome with a *P* value ≤ .20 were selected. Note that for this analysis because the 3 tobacco-related variables were significantly associated with the outcome in the univariate analyses, only “Smoking status (active or history of)” was selected for the multivariate analysis. A backward variable selection based on *P* values was then performed. The resulting multivariate model is displayed.

**Table E8:** Predictive factors of an incomplete test: Univariate analysis∗

Characteristic	N	Event n	Odds ratio	95% CI	*P* value
Age	1414	234	0.97	0.96-0.99	**<.001**
Gender	1414	234			.54
Female			–	–	
Male			0.88	0.61-1.32	
Smoking status, active or history of	1414	234	0.24	0.17-0.34	**<.001**
History of smoking	1414	234	0.27	0.18-0.40	**<.001**
Active smoking	1414	234	0.37	0.20-0.64	**<.001**
Insulin-dependent diabetes	1414	234	0.83	0.55-1.22	.35
Hypertension	1414	234	1.03	0.76-1.42	.83
No. of distal anastomoses, total	1414	234			**<.001**
1-2			–	–	
3			2.75	1.82-4.23	
4-6			4.37	2.97-6.57	
No. SV	1414	234			**<.001**
0			–	–	
1-2			0.19	0.12-0.28	
No. LITA	1414	234			**<.001**
0-1			–	–	
2-3			2.55	1.92-3.39	
No. RITA	1414	234			**<.001**
0-1			—	—	
2-4			6.35	4.70-8.64	
LAD flow, unrevised	1356	176	0.98	0.97-0.98	**<.001**

Figures in boldface are statistically significant. *SV*, Saphenous vein; *LITA*, left internal thoracic artery; *RITA*, right internal thoracic artery; *LAD*, left anterior descending artery.

∗ The following variables were evaluated as predictors of incomplete test: age; gender; active smoking; smoking status (active or history of); insulin-dependent diabetes; hypertension; off-pump surgery; number of distal anastomoses (total); number SVG; Number LITA; Number RITA; Number RA; Initial LAD flow. The predictors were evaluated through a logistic regression model (on observed cases). Graphs were also computed to display the association between the potential predictors and the incomplete/complete status (boxplots for continuous variables and bar plots of frequency for categorical variables). Note that number of RA was not evaluated because of the low number of cases; for the same reason, for several variables some levels were regrouped. The contingency tables show the row number of cases. Off pump surgery was not evaluated due to its systematic association to being tested. Univariate analyses for prediction of incomplete testing (all bypass tested vs at least 1 bypass not tested).

**Table E9:** Multivariate analysis of incomplete testing∗

Characteristic	Event n	Odds ratio	95% CI	*P* value
Age	234	0.98	0.96-0.99	**.007**
Smoking status, active or history of	234			**<.001**
No		–	–	
Yes		0.43	0.29-0.63	
No. of distal anastomoses (total)	234			**<.001**
1-2		–	–	
3		2.86	1.66-4.95	
4-6		3.03	1.60-5.74	
No. of SV	234			**<.001**
0		–	–	
1-2		0.25	0.14-0.42	
No. of RITA	234			**.017**
0-1		–	–	
2-4		1.87	1.12-3.15	

Figures in boldface are statistically significant. *SV*, Saphenous vein; *RITA*, right internal thoracic artery.

∗ Variables associated to the outcome with a *P* value ≤ .20 were selected. Note that for this analysis because the 3 tobacco-related variables were significantly associated with the outcome in the univariate analyses, only “Smoking status, active or history of” was selected for the multivariate analysis. A backward variable selection based on *P* values was then performed. The resulting multivariate model is displayed. Multivariate analysis for prediction of incomplete testing (all bypass tested vs at least one bypass not tested).

## References

[1] Gaudino M., Franco A., Bhatt D.L., Alexander J.H., Abbate A., Azzalini L. (2021). The association between coronary graft patency and clinical status in patients with coronary artery disease. Eur Heart J.

[2] Harik L., Sandner S., Gaudino M. (2023). Unanswered questions on coronary artery graft patency and clinical outcomes. Curr Opin Cardiol.

[3] Kieser T.M., Rose S., Kowalewski R., Belenkie I. (2010). Transit-time flow predicts outcomes in coronary artery bypass graft patients: a series of 1000 consecutive arterial grafts. Eur J Cardio Thorac Surg.

[4] Jokinen J.J., Werkkala K., Vainikka T., Perakyla T., Simpanen J., Ihlberg L. (2011). Clinical value of intra-operative transit-time flow measurement for coronary artery bypass grafting: a prospective angiography-controlled study. Eur J Cardio Thorac Surg.

[5] Lehnert P., Moller C.H., Damgaard S., Gerds T.A., Steinbruchel D.A. (2015). Transit-time flow measurement as a predictor of coronary bypass graft failure at one year angiographic follow-up. J Card Surg.

[6] Sousa-Uva M., Neumann F.-J., Ahlsson A., Alfonso F., Banning A.P., Benedetto U. (2019). 2018 ESC/EACTS Guidelines on myocardial revascularization. Eur J Cardio Thorac Surg.

[7] Kieser T.M., Taggart D.P. (2018). The use of intraoperative graft assessment in guiding graft revision. Ann Cardiothorac Surg.

[8] Singh S.K., Desai N.D., Chikazawa G., Tsuneyoshi H., Vincent J., Zagorski B.M. (2010). The graft imaging to improve patency (GRIIP) clinical trial results. J Thorac Cardiovasc Surg.

[9] Quin J.A., Noubani M., Rove J.Y., Krstacic J.E., Hattler B., Collins J.F. (2021). Veterans Affairs Randomized On/Off Bypass Follow-up Study (ROOBY-FS) Group. Coronary artery bypass grafting transit time flow measurement: graft patency and clinical outcomes. Ann Thorac Surg.

[10] Di Giammarco G., Pano M., Cirmeni S., Pelini P., Vitolla G., Di Mauro M. (2006). Predictive value of intraoperative transit-time flow measurement for short-term graft patency in coronary surgery. J Thorac Cardiovasc Surg.

[11] Gaudino M., Sandner S., Di Giammarco G., Di Franco A., Arai H., Asai T. (2021). The use of intraoperative transit time flow measurement for coronary artery bypass surgery. Circulation.

[12] Becit N., Erkut B., Ceviz M., Unlu Y., Colak A., Kocak H. (2007). The impact of intraoperative transit time flow measurement on the results of on-pump coronary surgery. Eur J Cardio Thorac Surg.

[13] Bauer S.F., Bauer K., Ennker I.C., Rosendahl U., Ennker J. (2005). Intraoperative bypass flow measurement reduces the incidence of postoperative ventricular fibrillation and myocardial markers after coronary revascularisation. Thorac Cardiovasc Surg.

[14] Taggart D.P., Thuijs D.J.F.M., Di Giammarco G., Puskas J.D., Wendt D., Trachiotis G.D. (2020). Intraoperative transit-time flow measurement and high-frequency ultrasound assessment in coronary artery bypass grafting. J Thorac Cardiovasc Surg.

[15] Taggart D.P., Altman D.G., Gray A.M., Lees B., Gerry S., Benedetto U. (2016). Randomized trial of bilateral versus single internal-thoracic-artery grafts. N Engl J Med.

[16] Laali M., Nardone N., Demondion P., D'Alessandro C., Guedeney P., Barreda E. (2022). Impact of transit-time flow measurement on early postoperative outcomes in total arterial coronary revascularization with internal thoracic arteries: a propensity score analysis on 910 patients. Interact Cardiovasc Thorac Surg.

[17] Herman C., Sullivan J.A., Buth K., Legare J.F. (2008). Intraoperative graft flow measurements during coronary artery bypass surgery predict in-hospital outcomes. Interact Cardiovasc Thorac Surg.

[18] Thuijs D.J.F.M., Bekker M.W.A., Taggart D.P., Kappetein A.P., Kieser T.M., Wendt D. (2019). Improving coronary artery bypass grafting: a systematic review and meta-analysis on the impact of adopting transit-time flow measurement. Eur J Cardio Thorac Surg.

[19] Chaban R., Buschmann K., Dohle D.S., Schnelle N., Vahl C.F., Ghazy A. (2021). Training cardiac surgeons: safety and requirements. Semin Thoracic Surg.

[20] Silva M., Rong L.Q., Naik A., Rahouma M., Hameed I., Robinson B. (2020). Intraoperative graft flow profiles in coronary artery bypass surgery: a meta-analysis. J Card Surg.

[21] Krasopoulos G., D’Alessio A., Verdichizzo D., Muretti M., Turton M.J., Gerry S. (2020). Beyond patency: functional assessment of adequacy using internal mammary artery grafting to the left anterior descending artery. J Card Surg.

